# Cancer-Derived Exosomal miR-651 as a Diagnostic Marker Restrains Cisplatin Resistance and Directly Targets ATG3 for Cervical Cancer

**DOI:** 10.1155/2021/1544784

**Published:** 2021-09-15

**Authors:** Xiaofan Zhu, Ling Long, He Xiao, Xuan He

**Affiliations:** ^1^Department of Obstetrics and Gynecology, Chongqing General Hospital, Chongqing 401147, China; ^2^Department of Obstetrics and Gynecology, The Second Affiliated Hospital of Army Medical University, Chongqing 400037, China; ^3^Cancer Center, Daping Hospital, Army Medical University, Chongqing 400010, China

## Abstract

**Objective:**

Cancer-derived exosomes can facilitate drug resistance in cervical cancer. However, the mechanisms remain elusive. Herein, we observed the roles of exosomal miR-651 in cisplatin resistance of cervical cancer.

**Methods:**

Circulating miR-651 was detected in cervical cancer and healthy individuals. The diagnostic efficacy was determined. When transfected with miR-651 mimics, cisplatin resistance, apoptosis, and proliferation were assessed. The cancer-derived exosomes were separated and identified. We observed the uptake of PKH67-labeled exosomes by HeLa/S cells. After coculture with exosomes secreted by HeLa/S or HeLa/DDP cells, malignant behaviors were examined in HeLa/S cells. The interactions between ATG3 and miR-651 were validated by dual luciferase report. Biological behaviors were investigated for HeLa/S cells cocultured with exosomes secreted by miR-651 mimic-transfected HeLa/DDP cells.

**Results:**

Downregulated circulating miR-651 was found in cancer subjects than healthy individuals. It possessed high sensitivity and accuracy in diagnosing cervical cancer (AUC = 0.9050). Lower miR-651 expression was confirmed in HeLa/DDP than HeLa/S cells. Forced miR-651 lessened cisplatin resistance and proliferation and elevated apoptosis in HeLa cells. ATG3 was a direct target of miR-651. The exosomes isolated from HeLa cells were rich in CD63, CD9, and CD81 proteins, thereby identifying the isolated exosomes. Exosomes secreted by HeLa/DDP cells can be absorbed by HeLa/S cells. When being cocultured with exosomes secreted by HeLa/DDP cells, malignant behaviors of HeLa/S cells were enhanced, which were ameliorated by miR-651 mimic exosomes.

**Conclusion:**

Our findings showed that cancer-derived exosomal miR-651 restrained cisplatin resistance and directly targeted ATG3, indicating that exosomal miR-651 could be a therapeutic agent.

## 1. Introduction

Cervical cancer is a major killer that seriously threatens women's health [1]. Although the application of cervical cancer vaccine can effectively reduce the incidence of cervical cancer, mortality as well as morbidity of this malignancy still ranks fourth among women worldwide [2]. The current treatment methods for cervical cancer are comprehensive such as surgery, chemotherapy, radiotherapy, and immunotherapy [3]. Nevertheless, not all subjects can achieve satisfactory outcomes, especially for subjects with advanced stages [4–6]. For locally advanced individuals, the current treatment strategy is neoadjuvant chemotherapy followed by surgery for chemotherapy-sensitive patients, while insensitive patients are directly converted to radiotherapy [7]. If there are indicators that can effectively predict the sensitivity of patients to chemotherapy, this part of the patients who are not sensitive to chemotherapy can save the time and expense of neoadjuvant chemotherapy and directly transfer to radiotherapy to obtain better outcomes [8]. Therefore, continuing to study the pathogenesis of cervical cancer, looking for specific therapeutic targets and markers for predicting chemotherapy sensitivity is still extremely critical for cervical cancer therapy.

Exosomes are extracellular vesicles with a diameter of 50-140 nm, which are formed by cells through regulation processes such as endocytosis, fusion, and efflux [9–11]. Exosomes carry signals between cells by transmitting small biologically active molecules substances like microRNAs (miRNAs) [12]. Exosomes can be separated from various body fluids and cell culture supernatants, and their function and composition are determined by their cell sources [13]. Exosomes are abundant in the microenvironment of malignant tumors, which are involved in the processes of invasion, metastases, angiogenesis, and chemotherapy resistance of malignant tumors [14, 15]. miRNA is an endogenous, noncoding single-stranded small RNA, with 19-23 nucleotides in length [16]. Mature miRNAs have the functions of inhibiting target mRNA expression after transcription [17]. Various miRNA markers have been identified for cervical cancer. Exosomal miRNAs have been promising players for cervical cancer [18]. Previous research has highlighted the impacts of miR-651 on non-small cell lung cancer [19] and nasopharyngeal carcinoma [20]. Unfortunately, it remains uncharted concerning miR-651 on cervical cancer. Here, our study identified circulating miR-651 as a diagnostic marker upon this malignancy. Moreover, forced exosomal miR-651 could restrain cisplatin resistance and directly target ATG3 for cervical cancer cells, suggesting the action of miR-651 on cervical cancer progress.

## 2. Materials and Methods

### 2.1. Patients and Specimens

From January 2018 to January 2019, 30 newly diagnosed cervical cancer patients were recruited in the Daping Hospital, Army Medical University. All patients were confirmed histopathologically. Meanwhile, 30 healthy age-matched individuals were selected. This study excluded cervical cancer subjects who had undergone surgery, radiotherapy, chemotherapy, etc. Furthermore, subjects with a history of severe organ disease or other systemic tumors were also excluded. All participants provided written informed consent. This study met the requirements of the Ethics Committee of Daping Hospital, Army Medical University (2018002). 5 mL whole blood samples were harvested from each subject by using EDTA anticoagulation tube. Following centrifugation and separation, samples were collected in an EP tube and stored at -80°C for later use.

### 2.2. Quantitative Real-Time Polymerase Chain Reaction (qRT-PCR)

Tissues or cells were lysed by 1 mL TRIzol (Invitrogen, USA) on ice for 10 min. The lysate was transferred to the EP tube. After adding 200 *μ*L chloroform, the sample stood for 5 min and was centrifuged at 12,000 g at 4°C for 30 min. The upper aqueous layer solution was pipetted and mixed with an equal volume of isopropanol. After centrifugation at 12,000 g for 15 min at 4°C, the sample was washed and precipitated with 85% ethanol. An appropriate amount of DEPC water was added to dissolve RNA. qRT-PCR was then carried out by SYBR Green fluorescence quantitative detection kit (Beyotime, Beijing, China). The primer sequences were as follows: miR-651—5′-TGGGTAAAGTGCTTATAGTGC3′ (forward) and 5′-CACCAGGGTCCGAGGT-3′ (reverse); ATG3—5′-GACCCCGGTCCTCAAGGAA-3′ (forward) and 5′-TGTAGCCCATTGCCATGTTGG-3′ (reverse); U6—5′⁃TGCGGGTGCTCGCTTCGGCAGC⁃3′ (forward) and 5′⁃CCAGTG⁃CAGGGTCCGAGGT⁃3′; and GAPDH—5′-CTGGGCTACACTGAGCACC-3′ (forward) and 5′-AAGTGGTCGTTGAGGGCAATG-3′ (reverse). Threshold cycle (Ct) was determined for each sample. The relative expression was determined using the 2^-∆∆Ct^ method.

### 2.3. Cell Culture

Human normal cervical epithelial cells (HcerEpic), C33A, HT-3, cisplatin (DDP)-sensitive cervical cancer cell line HeLa/S, and DDP-resistant cell line HeLa/DDP were purchased from Shanghai Institute of Biological Sciences, Chinese Academy of Sciences (China). These cells were grown on DMEM medium (Hyclone, USA) plus fetal bovine serum (FBS; Hyclone, USA) in a 37°C constant temperature and 5% CO_2_ incubator.

### 2.4. Transfection

HeLa cells were seeded on a 24-well plate (4 × 10^4^ cells/well). On the second day, the cells were transfected with miR-651 mimics (Genepharma, Shanghai, China), miR-651 inhibitors (Genepharma, Shanghai, China), or negative control (NC; Genepharma, Shanghai, China). 50 *μ*L serum-free medium Opti-MEM was used to dilute 1.25 *μ*L miR-651 mimics, miR-651 inhibitors, or NC at a concentration of 20 *μ*mol/L and incubated for 5 min at room temperature. Similarly, 1 *μ*L Lipofectamine™ 2000 was diluted with 50 *μ*L serum-free medium Opti-MEM and incubated at room temperature for 5 min. The diluted miR-651 mimics, miR-651 inhibitors, or NC were mixed with Lipofectamine™ 2000 and incubated for 20 s, which were then added to 24 plates. After culturing for 5 h, the mixed culture medium was removed and fresh culture medium was replaced, and then culturing was continued for 24 h.

### 2.5. Cell Counting Kit-8 (CCK-8)

HeLa cells were inoculated into 96-well plates (8 × 10^3^ cells/well). After continuing to incubate for 12 h, the DMEM medium was discarded, and 200 *μ*L DMEM medium was added containing 0, 50, 100, 200, 400, and 800 *μ*g/mL DDP. After 48 h, 20 *μ*L CCK-8 solution (Dojindo, Japan) was added to each well. After culturing for 2 h, the culture medium was discarded. The absorbance at 450 nm was measured with a multifunctional microplate reader. The half inhibitory concentrations (IC50) of DDP were determined on HeLa cells.

### 2.6. Flow Cytometry

The cells were trypsinized into a single cell suspension and washed with PBS. The cells were inoculated into a 6-well plate (2.0 × 10^5^/well). 5 *μ*L Annexin V-FITC and 5 *μ*L PI (Sigma, USA) were added, separately. After mixing thoroughly, the cells were incubated for 10 min at room temperature in the dark. The cells were tested on the flow cytometer (BD, Germany).

### 2.7. Colony Formation Assay

HeLa cells were trypsinized to prepare a cell suspension. After counting the cells, they were spread in a 6-well plate (300 cells/well). There were 3 multiple holes in each group. The cells were repeated pipetted and shaken to prevent the cells from forming clumps. After 2 weeks of culture, the cells were fixed with 4% paraformaldehyde and stained with 0.1% crystal violet (Sigma, USA). The number of colonies was counted under a microscope (Olympus, Japan).

### 2.8. Exosome-Free Serum Preparation

FBS was centrifuged at 100 000 g for 70 min, and the precipitate was removed to obtain exosome-free FBS. HeLa/S cells were cultured with RPMI 1640 medium (Hyclone, USA) with exosome-free 10% FBS.

### 2.9. Exosome Collection in Cell Culture Supernatant

Logarithmic growth of HeLa/S and HeLa/DDP cells was trypsinized and cultured in RPMI 1640 medium with 10% FBS without exosomes for 48 h. The cell culture supernatant of HeLa/S and HeLa/DDP cell culture medium was collected. After centrifugation at 500 g for 10 min and then at 12,000 g for 20 min, the supernatant was collected. After filtering through a 0.22 *μ*m pore filter, the supernatant was centrifuged at 100,000 g for 2 h and resuspended in phosphate-buffered saline (PBS) solution. After centrifugation at 100,000 g for 2 h, the precipitate was resuspended in PBS to form HeLa/S and HeLa/DDP exosomes, which were stored for later use.

### 2.10. Western Blot for CD63, CD9, and CD81

Western blot was utilized for detecting exosomal marker proteins including CD63, CD9, and CD81. 20 *μ*L resuspended exosomes were taken and added by 10 *μ*L protein lysis buffer. Following being lysed on ice for 30 min, samples were centrifuged at 12,000 g for 20 min. The supernatant was collected to obtain the exosomal protein. 80 *μ*g exosomal protein was under electrophoresis for 2 h and transferred to membranes. Then, membranes were blocked with skim milk powder for 1 h and incubated with corresponding primary antibodies containing CD63 (1/200; ab216130; Abcam, USA), CD9 (1/500; ab223052; Abcam, USA), and CD81 (1/1000; ab109201; Abcam, USA) overnight. Then, membranes were incubated with secondary antibody (1/2000; ab7090; Abcam, USA) for 1 h. After ECL (Absin, Shanghai, China) color development, dark room exposure, and development, we obtained protein bands through a gel imaging system.

### 2.11. Uptake of Exosomes

PKH67 is a membrane labeling dye that can bind to the lipid membrane of exosomes and emit green fluorescence, which can be used to identify the presence of exosomes. 100 *μ*L exosomes were incubated with 1 *μ*L PKH-26 dye, which were then incubated by 1 mL Diluent C, 200 *μ*L 1% BSA/PBS, and 3 *μ*L PKH-26 dye for 20 min. Under centrifugation at 100 000 g for 70 min, the PKH67-labeled exosome pellet was obtained. The HeLa/S cells were cultured in a 12-well plate. When they reached 70% confluence, they were replaced with a fresh medium containing PKH67-labeled exosomes and incubated for 24 h. After washing with PBS, the cells were fixed with paraformaldehyde (Sigma, USA) for 20 min. DAPI was used for staining the nucleus. The uptake of exosomes by HeLa/S cells was investigated under a confocal fluorescence microscope (Leica, Germany).

### 2.12. Western Blot for ATG3, LC3II/I, and p62

Tissues and cells were added with protein lysis buffer containing 100× protease and phosphatase inhibitors and repeatedly pipetted. After centrifugation, the supernatant was transferred to a new EP tube. BCA (Sigma, USA) measured the total protein concentration. After boiling and denaturing, the sample was loaded on 8% SDS-PAGE electrophoresis. The proteins were transferred to the PVDF membrane (Millipore, USA). The 5% skimmed milk powder was used for sealing membranes at room temperature for 2 h. After washing 3 times with TBST, membranes were incubated with primary antibodies containing ATG3 (1/500; ab233562; Abcam, USA), LC3I/II (1/1000; ab128025; Abcam, USA), p62 (1/1000; ab91526; Abcam, USA), and GAPDH (1/10000; ab181602; Abcam, USA) at 4°C overnight. Following washing with TBST, secondary antibody (1/10000; ab7090; Abcam, USA) incubation was presented for 2 h at room temperature. The bands were exposed by ECL chemiluminescence. GAPDH was utilized as an internal reference.

### 2.13. Dual Luciferase Report

The 3′-UTR sequence of miR-651 predicted target gene ATG3 was retrieved from the NCBI website. The ATG3 gene 3′-UTR plasmid pLUC-ATG3 was constructed synthetically (GenePharma, Shanghai, China). The HeLa/S and HeLa/DDP cells were, respectively, seeded in 24-well plates. According to the Lipofectamine™ 2000 instructions, 100 ng pLUC-ATG3 was cotransfected with 50 nmol/L miR-651 mimic or NC. After 48 h, the luciferase activity was detected by a dual luciferase reporter gene kit.

### 2.14. Statistical Analysis

The measurement data are expressed by the mean ± standard deviation. SPSS 18.0 software (SPSS Inc., USA) was utilized for statistical analysis. Comparisons between groups were performed through Student's *t* test or one-way ANOVA. Receiver operating characteristic curves (ROCs) were drawn, and area under the curve (AUC) was calculated for assessing the diagnostic potential of circulating miR-651 in cervical cancer. ATG3 expression was analyzed using the UALCAN database (http://ualcan.path.uab.edu/analysis.html). Pearson's analysis was presented between miR-651 and ATG3 in 30 pairs of cervical cancer and healthy individuals. *p* value < 0.05 indicated statistical significance.

## 3. Results

### 3.1. Circulating miR-651 as a Prognostic Marker for Cervical Cancer

**Figure 1:**
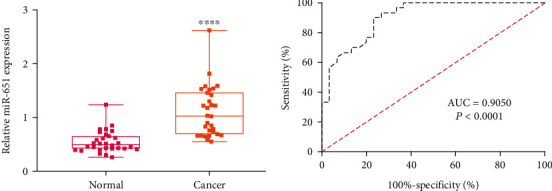
Expression and prognosis of circulating miR-651 in cervical cancer. (a) Downregulated circulating miR-651 in cervical cancer patients. Compared to normal, ^∗∗∗∗^*p* < 0.0001. (b) AUC curves for circulating miR-651 expression among cervical cancer subjects and healthy individuals.

This study recruited 30 cervical cancer patients and 30 healthy individuals. Circulating miR-651 expression was detected via RT-qPCR. Data suggested that lowered miR-651 expression was found in cervical cancer plasma than normal specimens (*p* < 0.0001; Figure 1(a)). We assessed the diagnostic potential of circulating miR-651 in cervical cancer. In Figure 1(b), the data showed that circulating miR-651 displayed a highly sensitive and accurate capacity for diagnosing cervical cancer (AUC = 0.9050, *p* < 0.0001). The above findings were indicative of circulating miR-651 as an underlying diagnostic marker of cervical cancer.

### 3.2. Downregulation of miR-651 in DDP-Resistant Cervical Cancer Cells

**Figure 2:**
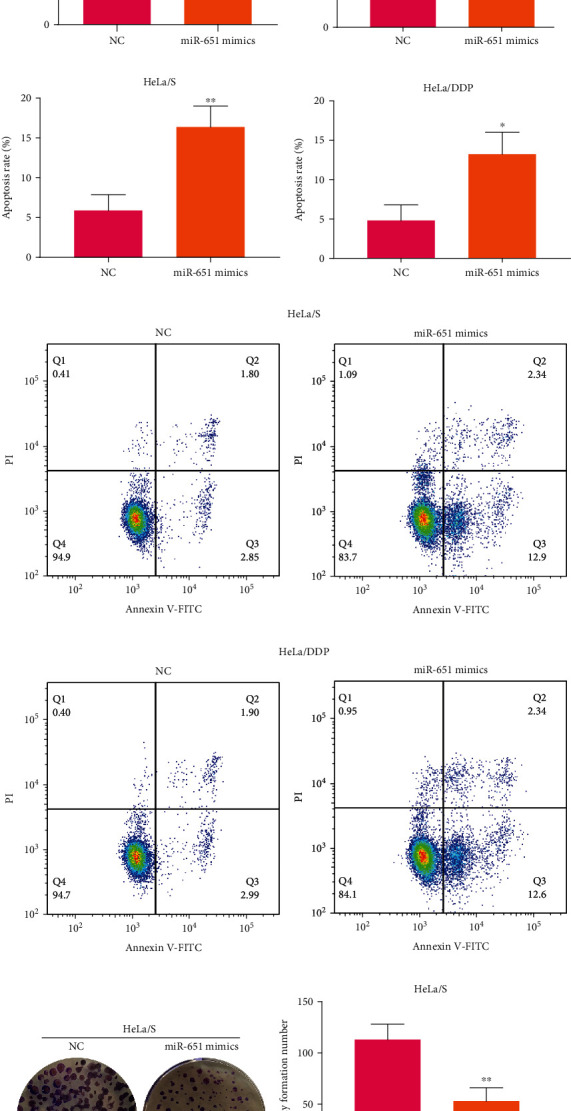
miR-651 expression and its functions on cisplatin resistance, proliferation, and apoptosis in cervical cancer cells. (a) Comparison of miR-651 expression in HcerEpic, C33A, HT-3, HeLa/S, and HeLa/DDP cell lines. (b) Verification of miR-651 expression in HeLa/S and HeLa/DDP cells with NC or miR-651 mimic transfection. (c, d) Determination of the IC50 values of cisplatin on NC- or miR-651 mimic-induced HeLa/S as well as HeLa/DDP cells. (e–h) Flow cytometry for apoptosis rates of HeLa/S and HeLa/DDP cells under treatment with NC or miR-651 mimics. (i–l) The colony formation number of NC- or miR-651 mimic-induced HeLa/S as well as HeLa/DDP cells. ^∗^*p* < 0.05; ^∗∗^*p* < 0.01; ^∗∗∗^*p* < 0.001; ^∗∗∗∗^*p* < 0.0001.

To investigate the roles of miR-651 on cisplatin resistance of cervical cancer, this study firstly examined its levels in cervical cancer cells. Its expression was compared in HcerEpic, C33A, HT-3, HeLa/S, and HeLa/DDP cell lines via RT-qPCR assay. As shown in Figure 2(a), in comparison to HcerEpic normal cervical epithelial cells, miR-651 downregulation was found in C33A (*p* < 0.01), HT-3 (*p* < 0.001), HeLa/S (*p* < 0.001), and HeLa/DDP (*p* < 0.0001) cervical cancer cells. More importantly, miR-651 exhibited reduced expression in HeLa/DDP cells than HeLa/S cells (*p* < 0.001). The above data suggested that downregulated miR-651 might participate in inducing cisplatin resistance of cervical cancer.

### 3.3. Forced miR-651 Exerts Inhibitory Action on Cisplatin Resistance and Proliferation and Motivates Apoptosis in Cervical Cancer Cells

Then, this study assessed whether forced miR-651 ameliorated cisplatin resistance of cervical cancer cells. HeLa/S as well as HeLa/DDP cells were separately transfected with miR-651 mimics. By RT-qPCR, miR-651 expression was determined. As expected, forced miR-651 expression was confirmed in HeLa/S and HeLa/DDP (both *p* < 0.0001) cell lines (Figure 2(b)). This demonstrated the successful elevation of miR-651 expression in two cells. miR-651 mimic- and NC-induced HeLa/S as well as HeLa/DDP cells were treated with a range of concentrations of DDP. The IC50 values were separately calculated in all groups of cells. In Figure 2(c), the reduction in IC50 values was detected in miR-651 mimic-induced HeLa/S cells (*p* < 0.01). Analogously, miR-651 mimics cut down IC50 values of HeLa/DDP cells (*p* < 0.0001; Figure 2(d)). Putting together, forced miR-651 could relieve cisplatin resistance of cervical cancer. The assessment of apoptotic levels was presented through flow cytometry. Data demonstrated that miR-651 mimic transfection elevated apoptosis rates of HeLa/S cells (*p* < 0.01; Figure 2(e)). The same findings were confirmed in HeLa/DDP cells (*p* < 0.05; Figure 2(f)). Hence, forced miR-651 expedited cervical cancer cellular apoptosis. The representative of flow cytometry for each group was exhibited in Figures 2(g) and 2(h). Following transfection with miR-651 mimics, colony formation number of HeLa/S cells was markedly reduced than NC transfection (*p* < 0.01; Figures 2(i) and 2(j)). Meanwhile, we counted colony formation number of HeLa/DDP cells. Likewise, the decrease in colony formation number was verified in HeLa/DDP cells with miR-651 mimic transfection (*p* < 0.01; Figures 2(k) and 2(l)). The above data suggested that forced miR-651 restrained proliferative capacity of cervical cancer cells.

### 3.4. Sensitive Cervical Cancer Cells Absorb Exosomes Secreted by Cisplatin-Resistant Cancer Cells

**Figure 3:**
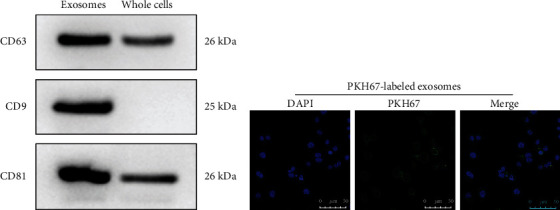
Sensitive cervical cancer cells absorb exosomes secreted by cisplatin-resistant cancer cells. (a) Western blot for exosomal markers including CD63, CD9, and CD81. (b) Detection of PKH67-labeld green fluorescence for HeLa/S cells. Bar = 50 *μ*m.

This study observed the functions of exosomes on cisplatin resistance in cervical cancer. We isolated exosomes in the culture supernatant of HeLa/DDP cells through high-speed centrifugation. To evaluate whether exosomes were successfully isolated, western blot was utilized for examining exosome surface biomarkers including CD63, CD9, and CD81 in white precipitate samples. In Figure 3(a), white precipitate samples exhibited CD63, CD9, and CD81 expression, which was indicative of the success isolation of exosomes. We then investigated whether HeLa/S cells possessed the functions of absorbing exosomes secreted by cisplatin-resistant cancer cells. Following coculturing cisplatin-resistant exosomes and HeLa/S cells, this study found that PKH67-labeled green fluorescence exhibited a uniform distribution in HeLa/S cellular cytoplasm (Figure 3(b)). These findings confirmed that exosomes secreted by cisplatin-resistant cancer cells can be absorbed by sensitive cervical cancer cells.

### 3.5. Cisplatin-Resistant Exosomes Facilitate Cisplatin Resistance and Proliferation and Inhibit Apoptosis in Cervical Cancer Cells

**Figure 4:**
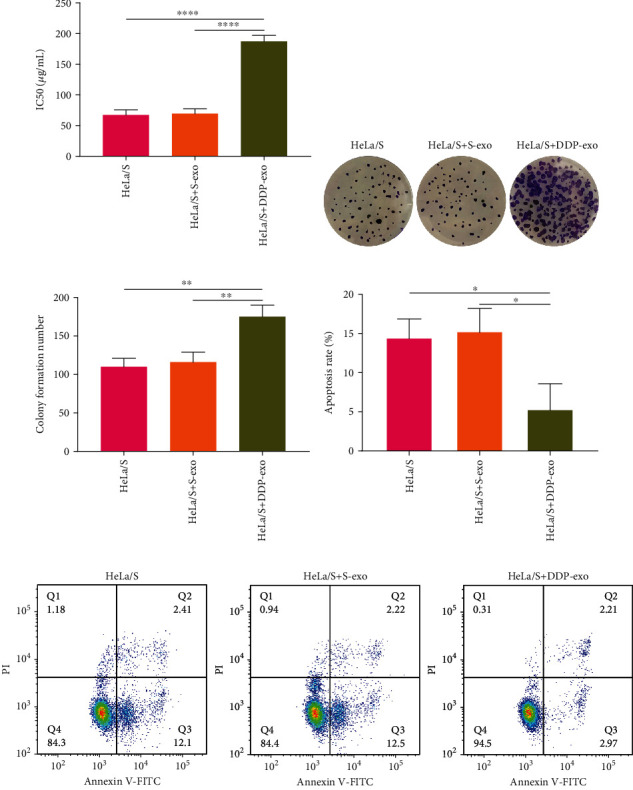
The functions of cisplatin-resistant exosomes on cisplatin resistance, proliferation, and apoptosis of cervical cancer cells. (a) Comparisons of the IC50 values of HeLa/S cells cocultured with exosomes secreted from HeLa/S or HeLa/DDP cells. (b, c) Evaluation of colony formation number of HeLa/S cells under coculture with exosomes from HeLa/S or HeLa/DDP cells. (d, e) Apoptosis levels of HeLa/S cells that were cocultured with exosomes from HeLa/S or HeLa/DDP cells. ^∗^*p* < 0.05; ^∗∗^*p* < 0.01; ^∗∗∗∗^*p* < 0.0001.

Here, we isolated exosomes from HeLa/S and HeLa/DDP cells, which were cocultured with HeLa/S cells. Following treatment with different concentrations of DDP lasting 48 h, we determined IC50 values for HeLa/S cell samples. In Figure 4(a), compared to HeLa/S cells, the IC50 values exhibited a distinct increase for those with cisplatin-resistant exosome coculture (*p* < 0.0001). Moreover, HeLa/S cells cocultured with cisplatin-resistant exosomes had the increase in IC50 values than those cocultured with exosomes secreted from HeLa/S cells (*p* < 0.0001). This indicated that DDP-resistant exosomes could induce drug resistance of cervical cancer. Then, we presented the assessment upon colony formation ability. Coculture of exosomes secreted from HeLa/DDP cells markedly heightened the colony formation capacities of HeLa/S cells (*p* < 0.01; Figures 4(b) and 4(c)). Meanwhile, compared to exosomes from HeLa/S cells, the increase in colony formation capacities of HeLa/S cocultured with exosomes from HeLa/DDP cells was confirmed (*p* < 0.01). Hence, DDP-resistant exosomes elevated proliferation of colony formation. As shown in flow cytometry, following coculture with exosomes from HeLa/DDP cells, apoptosis of HeLa/S cells was distinctly restrained (*p* < 0.05; Figures 4(d) and 4(e)). In comparison to coculture of exosomes from HeLa/S cells, there was a marked decrease in apoptosis of HeLa/S cells cocultured by exosomes from HeLa/DDP cells (*p* < 0.05). The above data suggested that cisplatin-resistant exosomes lowered apoptotic levels of cervical cancer cells.

### 3.6. Upregulation of ATG3 in Cervical Cancer

**Figure 5:**
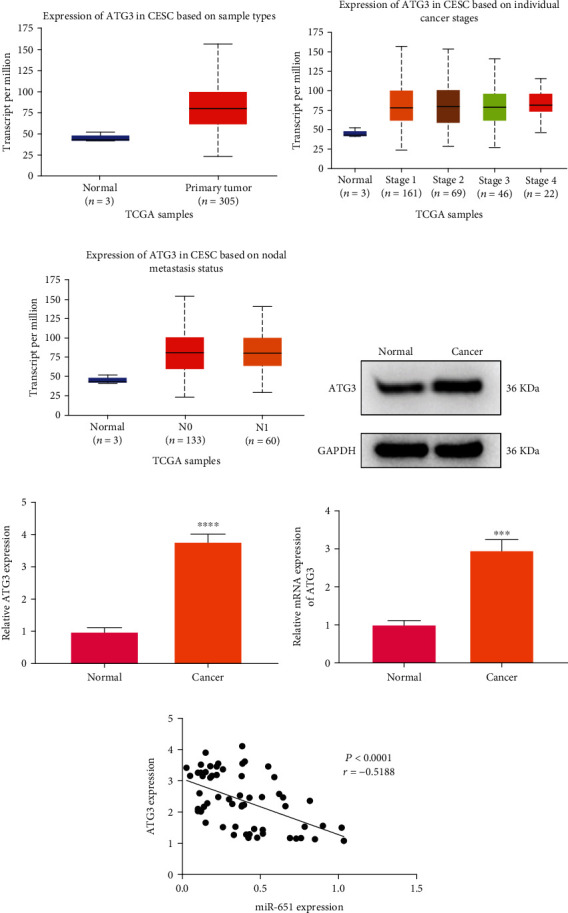
Upregulation of ATG3 in cervical cancer. (a) Box plot for ATG3 expression in cervical cancer and normal tissue specimens from TCGA database. (b) Comparisons of ATG3 expression in normal and different stages of cervical cancer tissues. (c) Expression of ATG3 in cervical cancer tissues based on nodal metastasis status. (d, e) Western blot for ATG3 expression in cervical cancer as well as normal plasma samples. (f) Expression of ATG3 in cervical cancer and normal plasma samples via RT-qPCR. (g) Correlation between circulating miR-651 and ATG3 among cervical cancer subjects. ^∗∗∗^*p* < 0.001; ^∗∗∗∗^*p* < 0.0001.

Following prediction, ATG3 was an underlying downstream target of cervical cancer. From TCGA database, we firstly assessed ATG3 expression in cervical cancer tissue specimens. Elevated ATG3 expression was found in primary tumors (*n* = 305) than normal tissues (*n* = 3), as shown in Figure 5(a) (*p* = 0.0389). Then, we compared the differences in ATG3 expression among different stages. As a result, compared with normal, stage 1 (*p* = 0.0410), stage 2 (*p* = 0.0409), and stage 4 (*p* = 0.0364) exhibited higher ATG3 expression (Figure 5(b)). Meanwhile, our data showed that in comparison to normal, tissues that had no regional lymph node metastasis (N0; *p* = 0.0444) or metastases in 1 to 3 axillary lymph nodes (N1; *p* = 4.3465*e* − 07) displayed increased ATG3 expression (Figure 5(c)). Also, we detected ATG3 expression in plasma samples from cervical cancer and normal individuals. Western blot data confirmed the upregulation of ATG3 proteins in cervical cancer than normal samples (*p* < 0.0001; Figures 5(d) and 5(e)).  Figure 5(f) shows that circulating ATG3 had increased expression in cervical cancer plasma than normal specimens (*p* < 0.001). Correlation analysis revealed that miR-651 exhibited a negative interaction with ATG3 among cervical cancer subjects (*p* < 0.0001, *r* = −0.5188; Figure 5(g)).

### 3.7. ATG3 As a Direct Target of miR-651 in Cervical Cancer

**Figure 6:**
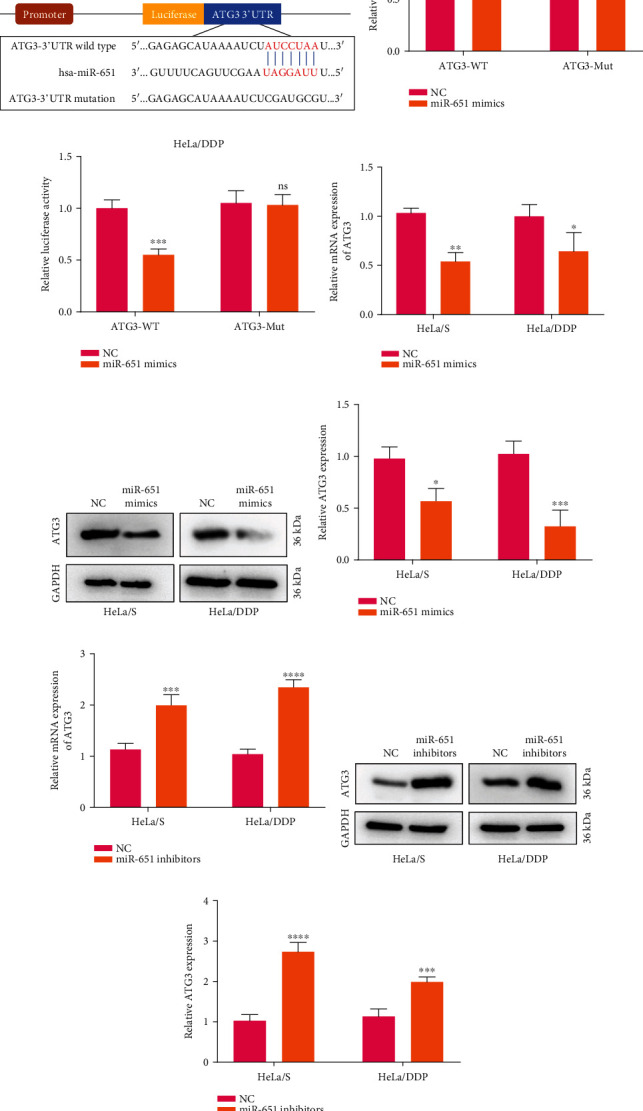
ATG3 as a direct target of miR-651 in cervical cancer. (a) Binding sites between miR-651 and wild-type or mutated ATG3. (b, c) Dual luciferase report for confirming the interactions between miR-651 and ATG3 in HeLa/S and HeLa/DDP cells. (d–f) ATG3 mRNA and protein expression in HeLa/S and HeLa/DDP cells transfected with miR-651 mimics or NC. (g–i) Expression of ATG3 mRNA and protein in HeLa/S and HeLa/DDP cells with miR-651 inhibitor or NC transfection. ^∗^*p* < 0.05;  ^∗∗^*p* < 0.01;  ^∗∗∗^*p* < 0.001;  ^∗∗∗∗^*p* < 0.0001.

To confirm the interactions between miR-651 and ATG3, dual luciferase report was presented (Figure 6(a)). Under ATG3-WT-induced HeLa/S cells, miR-651 mimics significantly lessened luciferase activity than NC (*p* < 0.001; Figure 6(b)). However, miR-651 mimics did not change luciferase activity in ATG3-Mut-induced HeLa/S cells. Similar findings were found in HeLa/DDP cells (Figure 6(c)). Thus, ATG3 may be a direct target of miR-651 in cervical cancer. This study then evaluated whether miR-651 impacted ATG3 expression in cervical cancer cells. In Figure 6(d), data showed that miR-651 mimics significantly decreased ATG3 mRNA expression both in HeLa/S (*p* < 0.01) and HeLa/DDP cells (*p* < 0.05). Meanwhile, ATG3 expression was detected by western blot. Figures 6(e) and 6(f) show that the reduction in ATG3 expression was detected in HeLa/S (*p* < 0.05) and HeLa/DDP cells (*p* < 0.001) with miR-651 mimic transfection than NC. After transfection with miR-651 inhibitors, ATG3 mRNA expression displayed an increased expression in comparison to NC in HeLa/S (*p* < 0.001) and HeLa/DDP cells (*p* < 0.0001; Figure 6(g)). Meanwhile, ATG3 expression was assessed through western blot. We found that compared to NC transfection, miR-651 inhibitors elevated ATG3 expression both in HeLa/S (*p* < 0.0001) and HeLa/DDP cells (*p* < 0.001; Figures 6(h) and 6(i)). Putting together, ATG3 was a target of miR-651 and forced miR-651 may lessen ATG3 expression in cervical cancer.

### 3.8. Exosome-Shuttled miR-651 Restrains Cisplatin Resistance and Proliferation and Facilitates Apoptosis in Cervical Cancer Cells

**Figure 7:**
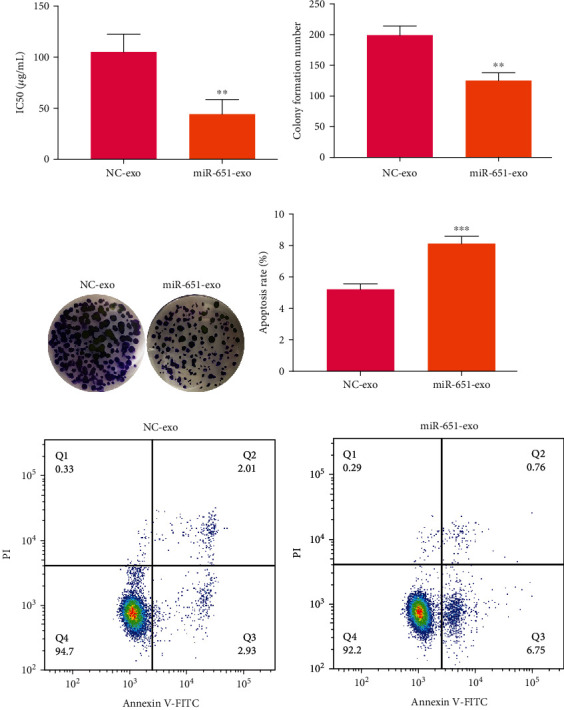
The cisplatin resistance, proliferation, and apoptosis of HeLa/S cells following coculturing with HeLa/DDP cells transfected by miR-651 mimics. (a) The IC50 values of HeLa/S cells that were cocultured with miR-651 mimic-transfected HeLa/DDP cells. (b, c) Colony formation number of HeLa/S cells after coculturing with miR-651 mimic-transfected HeLa/DDP cells. (d, e) Apoptotic levels of HeLa/S cells under coculture with miR-651 mimic-transfected HeLa/DDP cells. ^∗∗^*p* < 0.01; ^∗∗∗^*p* < 0.001.

It has been reported that cisplatin resistant exosomes may promote drug resistance of cervical cancer cells [21]. Here, this study investigated the roles of exosomal miR-651 upon cervical cancer cells. HeLa/DDP cells transfected by miR-651 mimics were cocultured with HeLa/S cells lasting 24 h. Then, we determined the IC50 values of HeLa/S cells. Our data showed that compared to NC, the reduction in the IC50 values was detected in HeLa/S cells that were cocultured with HeLa/DDP cells transfected by miR-651 mimics (*p* < 0.01; Figure 7(a)). Furthermore, this study found that the coculture model exhibited the decrease in colony formation number in comparison to NC group (*p* < 0.01; Figures 7(b) and 7(c)). Also, the apoptotic levels of HeLa/S cells were elevated following coculturing with HeLa/DDP cells transfected by miR-651 mimics (*p* < 0.001; Figures 7(d) and 7(e)). Taken together, exosome-shuttled miR-651 restrained cisplatin resistance and proliferation and facilitated apoptosis in cervical cancer cells.

### 3.9. Exosome-Shuttled miR-651 Lessens ATG3 Expression in Cervical Cancer Cells

**Figure 8:**
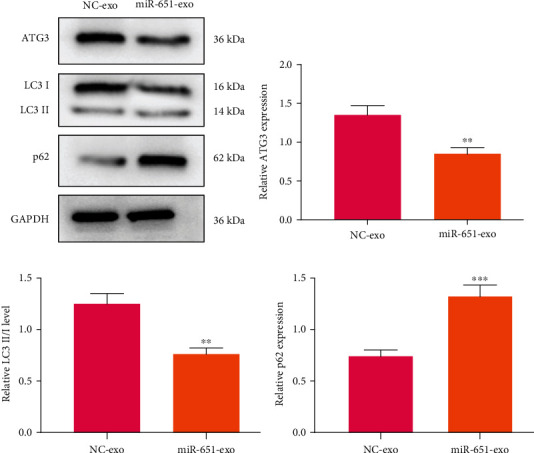
Expression of autophagy-related proteins including ATG3, LC3II/I and p62 in HeLa/S cells that were cocultured with HeLa/DDP cells transfected with miR-651 mimics. (a) Representative of western blot results. Quantitative results of (b) ATG3, (c) LC3II/I, and (d) p62 in HeLa/S cells following coculture with HeLa/DDP cells transfected with miR-651 mimics. ^∗∗^*p* < 0.01;  ^∗∗∗^*p* < 0.001.

**Figure 9:**
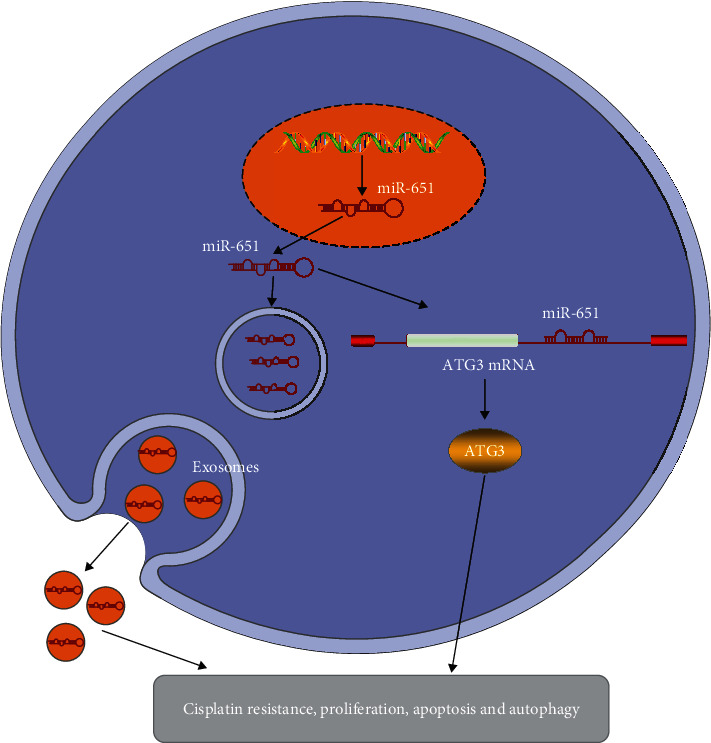
The schematic diagram of exosomal miR-651/ATG3 on cervical cancer progression.

Following coculture with miR-651 mimic-transfected HeLa/DDP cells, we examined ATG3 expression in HeLa/S cells. Data showed that ATG3 displayed the decreased expression in HeLa/S cells that were cocultured with HeLa/DDP cells transfected with miR-651 mimics (*p* < 0.01; Figures 8(a) and 8(b)). Furthermore, other autophagy-related proteins including LC3II/I and p62 were determined. The results demonstrated that LC3II/I levels were reduced (*p* < 0.01) and p62 expression (*p* < 0.001) was enhanced in HeLa/S cells that were cocultured with miR-651 mimic-transfected HeLa/DDP cells (Figures 8(c) and 8(d)). The schematic diagram of exosomal miR-651/ATG3 on cervical cancer is depicted in Figure 9.

## 4. Discussion

This study identified circulating miR-651 was downregulated in cervical cancer, which could be utilized as a marker for diagnosing this malignancy. Cancer-derived exosomal miR-651 may ameliorate cisplatin resistance and malignant progress of cervical cancer, highlighting exosomal miR-651 as a therapeutic agent against cervical cancer.

The miRNAs carried by exosomes are highly conservative and stable, which have been considered diagnostic markers or therapeutic agents in cervical cancer [22]. Zheng et al. confirmed exosomal let-7d-3p and miR-30d-5p as markers for diagnosing cervical cancer [23]. Konishi et al. confirmed that exosomal miR-22 could become a promising drug delivery system concerning cervical cancer radiotherapies [24]. Ma et al. proposed a circulating miRNA-signature containing miR-146a-5p, miR-151a-3p, miR-2110, and miR-21-5p upon cervical cancer diagnosis [25]. This study confirmed the downregulation of circulating miR-651 in cervical cancer. The AUC was 0.9050, suggesting that circulating miR-651 could be a sensitive and accurate diagnostic marker for cervical cancer. Its diagnostic value will be validated in a cohort with a larger sample size. Intriguingly, lower miR-21-5p expression was confirmed in HeLa/DDP than HeLa/S cells and forced its expression reduced IC50 values of DDP, indicating that miR-21-5p downregulation was in relation to cisplatin resistance. Previously, Shi et al. found that miR-144 may ameliorate cisplatin resistance of cervical cancer through LHX2 [26]. Yang et al. proposed that miR-497 could modulate cisplatin chemosensitivity of this malignancy through transketolase [27]. These findings highlighted the implications of miRNAs on drug resistance. Furthermore, our data showed that forced miR-651 induced apoptosis and weakened proliferation of HeLa cells, indicating miR-651 as a tumor suppressor gene.

In this study, ultracentrifugation was used to successfully isolate the exosomes of HeLa/S and HeLa/DDP cells. HeLa/S cells may absorb the exosomes secreted by HeLa/DDP cells, consistently with previous research [21]. We found that HeLa/DDP cell-derived exosomes can promote the proliferation and inhibit their apoptosis in HeLa/S cells, indicating that the exosomes secreted by HeLa/DDP cells may induce malignant transformation. Meanwhile, exosomes secreted from HeLa/DDP cells induced cisplatin resistance of HeLa/S cells. A previous study demonstrated that miR-106a/b from cisplatin resistant liver cancer cells may facilitate cisplatin resistance in cervical cancer cells [28]. Here, we found that exosomes secreted from miR-651 mimic-transfected HeLa/DDP cells weakened proliferation and lowered apoptotic levels for HeLa/S cells. ATG3 was a direct target of miR-651 in cervical cancer. MiR-651 carried by exosomes lessened ATG3 expression of HeLa/S cells. Chen et al. developed an autophagy-related model that can be predictive of cervical cancer subjects' outcomes [29]. Suppression of autophagy may weaken the progress of this malignancy [30]. Thus, cancer-derived exosomal miR-651 could restrain malignant behaviors of cervical cancer cells through ATG3. Previously, targeting autophagy could ameliorate metastasis and chemoresistance for various malignancies [31–33]. Furthermore, exosomes secreted from miR-651 mimics-transfected HeLa/DDP cells restrained the autophagy of HeLa/S cells. These data indicated that exosomal miR-651 ameliorated drug resistance of cervical cancer by lessening autophagy. However, several limitations of our study should be pointed out. Firstly, the clinical significance of exosomal miR-651 will be verified in a larger cervical cancer cohort. Second, the role of exosomal miR-651 on cervical cancer progression should be validated in vivo.

## 5. Conclusion

Collectively, this study showed that cancer-derived exosomal miR-651 may restrain cisplatin resistance and progression and directly targeted ATG3 in cervical cancer. Hence, exosomal miR-651 could be a therapeutic agent against cervical cancer.

## Data Availability

The datasets analyzed during the current study are available from the corresponding author on reasonable request.

## Abbreviations

miRNAs: MicroRNAs
qRT-PCR: Quantitative real-time polymerase chain reaction
Ct: Threshold cycle
DDP: Cisplatin
FBS: Fetal bovine serum
CCK-8: Cell counting kit-8
IC50: Half inhibitory concentration
ROCs: Receiver operating characteristic curves
AUC: Area under the curve.
